# A comparative study of the gastric ossicles of Trichodactylidae crabs (Brachyura: Decapoda) with comments on the role of diet and phylogeny in shaping morphological traits

**DOI:** 10.7717/peerj.5028

**Published:** 2018-06-19

**Authors:** Débora A. Carvalho, Pablo A. Collins, Renata Lima-Gomes, Célio Magalhães, Maria Victoria Torres, Verónica Williner

**Affiliations:** 1 Instituto Nacional de Limnología, Consejo Nacional de Investigaciones Científicas y Técnicas, Universidad Nacional del Litoral, Santa Fe, Argentina; 2 Facultad de Bioquímica y Ciencias Biológicas, Universidad Nacional del Litoral, Santa Fe, Argentina; 3 Instituto Nacional de Pesquisas da Amazônia, Manaus, Amazonas, Brazil; 4 Facultad de Humanidades y Ciencias, Universidad Nacional del Litoral, Santa Fe, Argentina

**Keywords:** Omnivore, Gastric teeth, Digestion, Freshwater crabs, Stomach content, Functional morphology

## Abstract

The gastric armature of decapod foregut is a feeding structure that sparks controversial debates about the role dietary and historical components have in shaping its morphological traits. Having previous information about the natural diet is an interesting way to gather evidence on this issue. For the present study, we analyzed the morphological traits of gastric ossicles involved directly in the maceration of food in nine species of freshwater crabs of the family Trichodactylidae (Brachyura: Decapoda) representing five genera, three tribes and two subfamilies. The analyzed gastric ossicles were quite consistent among closely related species, suggesting that the observed traits had a clear phylogenetic component. However, it was also noted that the morphological traits of the gastric teeth of trichodactylid crabs match well with the natural diet and presented likeness with general features of other species with a similar trophic habit. We discuss the influence of phylogeny and function on the design of morphological traits and propose to quantify the role of phylogeny and function in shaping morphological traits through the analysis of phylogenetic signals.

## Introduction

From benthopelagic isopods to land coconut crabs, crustaceans exhibit an extraordinary variety of body plans and occupy a very wide diversity of habitats (Watling & Thiel, 2015). Arguably, many of the lifestyles currently observed in crustaceans are a result of the morphological diversity shaped by the evolution and a body plan less constrained than other arthropods (Schram, 2013; Watling & Thiel, 2015). Attending to this, the ability to handle and process wide spectra of food types, product of morphological changes of the cephalic region and further specializations to grasp and manipulate food (Waloszek et al., 2007), aid the crustaceans to occupy and survive successfully in almost all types of habitats (Watling, 2013). Much of what we know about the anatomy of the crustacean group is the result of detailed studies of 19th century naturalists (Milne Edwards, 1834; Huxley, 1880; Mocquard, 1883) that stimulate the interest of more contemporary researchers in understanding the relation between form and function of such an amazing group (see Felgenhauer & Abele, 1985). In particular, the morphology of feeding structures of crustacean decapods still attracts the attention of crustacean biologists that intend to elucidate the role of phylogeny and diet in shaping their intricate traits (Caine, 1975; Felgenhauer & Abele, 1983; Felgenhauer & Abele, 1985; Allardyce & Linton, 2010).

Decapoda is the most diverse order of Crustacea with about 14,756 extant species (De Grave et al., 2009). The majority of decapod crustaceans live in aquatic environments and are able to cope with a broad variety of food types of different sizes, hardness and nutritional quality (Watling, 2013; Saborowski, 2015). The efficient exploitation of available or new trophic resources, together with the evolution of morphological traits, played a selective advantage to the diversification within Crustacea (Poore et al., 2017). In this way, it would be a simple and probably incorrect explanation to propose that trophic aspects play a reduced role in the evolutionary changes of morphological features (Waloszek et al., 2007). The gastric armature of the decapod foregut is an example of a feeding structure with a controversial debate about the role of dietary (Schaefer, 1970; Caine, 1975; Allardyce & Linton, 2010) and historical components (Dall & Moriarty, 1983; Felgenhauer & Abele, 1985; Felgenhauer & Abele, 1989; Brösing & Türkay, 2011) in shaping morphological traits.

The foregut of decapods is a complex and well-studied structure formed by three regions: the esophagus, and the cardiac (anterior) and pyloric (posterior) chambers. Once the food passes through the esophagus, it is stored in the cardiac chamber, the most spacious compartment of the stomach (Maynard & Dando, 1974; Meiss & Norman, 1977; Felgenhauer & Abele, 1989) and the one with the most interspecific variation (Icely & Nott, 1992). Although its general organization is similar among decapods, the complexity of the ossicle system varies within the infraorders of Decapoda (Icely & Nott, 1992). This variability is particularly noteworthy in those ossicles directly involved in the food fragmentation (i.e., the gastric mill and the cardiopyloric valve). According to Maynard & Dando (1974), among the gastric mill ossicles there are three dentate ossicles with direct functions in the maceration of food: the zigocardiac ossicles (paired), the urocardiac ossicle (unpaired), and the pterocardiac ossicles (paired). From these ossicles protrude the lateral, medial and accessory teeth, respectively. The cardiopyloric valve can also function as a masticatory ossicle, as well as a selective barrier between the cardiac and pyloric portions of the stomach (Caine, 1975).

Functional studies often focus in the correspondence between the morphology of these ossicles and the feeding habit (Caine, 1975; Kunze & Anderson, 1979; Salindeho & Johnston, 2003; Allardyce & Linton, 2010). A typical trait of carnivorous species is a gastric mill with blunt and smooth structures suitable to break down soft and less fibrous items (Heeren & Mitchell, 1997; Salindeho & Johnston, 2003; Allardyce & Linton, 2010). Conversely, herbivorous species require structures capable to disrupt the vegetal structure, exhibiting sharp ridges and cusps (Giddins et al., 1986; Allardyce & Linton, 2010). Omnivorous crabs should display intermediate morphological traits of herbivorous and carnivorous crabs (Allardyce & Linton, 2010; Carvalho et al., 2017). Although these studies provide evidence that morphological traits correspond to the trophic habit, some authors support the opposite, attributing a stronger phylogenetic weight to alimentary features (Caine, 1975; Felgenhauer & Abele, 1983; Felgenhauer & Abele, 1985; Felgenhauer & Abele, 1989; Brösing & Türkay, 2011).

The study of species with published information about their natural diet is one possibility to analyse the relationship between the trophic habit and gastric mill morphology. The Trichodactylidae is a family of freshwater brachyuran crabs of wide distribution (from Mexico to Argentina) that inhabit mostly the lowland rivers with Atlantic slope (Magalhães, 2003; Cumberlidge, 2016; Tadashi & Cumberlidge, 2016). Previous studies have found that trichodactylid crabs are mainly generalist and omnivorous with different importance of animal and vegetal items according to the species (Williner & Collins, 2002; Collins, Williner & Giri, 2007; Williner & Collins, 2013; Carvalho, 2014; Williner, Carvalho & Collins, 2014). For the present study, we analyzed the morphological traits of the gastric ossicles directly involved in the maceration of food (zigocardiac, urocardiac and pterocardiac ossicles and the cardiopyloric valve) of nine species of trichodactylid crabs representing five genera, three tribes and two subfamilies, according to the systematic classification of Magalhães & Türkay (1996). Among these species, the trophic habit of four of them (*Dilocarcinus pagei*, *Trichodactylus borellianus*, *Trichodactylus kensleyi*, *Trichodactylus fluviatilis*) (Williner & Collins, 2002; Williner & Collins, 2013; Carvalho, 2014; Williner, Carvalho & Collins, 2014; Segadilha & Silva-Soares, 2015; Costa et al., 2016; Nogueira-Costa et al., 2016) plus a fifth species in representation of the genus *Zilchiopsis* (*Zilchiopsis oronensis*) (V Williner, 2010, unpublished data) was already known. These five species cover the three tribes of Trichodactylidae. We expected to find similar morphological traits in species phylogenetically closer (Magalhães & Türkay, 1996) and a consistent relationship between morphology and diet. Previous data on the natural diet of some of these species helped us to understand the relationship between morphology, phylogeny and function.

## Materials and Methods

### Species of trichodactylid crabs

The specimens used in the present study are part of the collection of INALI (Instituto Nacional de Limnología, Santa Fe, Argentina) and INPA (Instituto Nacional de Pesquisas da Amazônia, Manaus, Brazil). We observed the stomachs of nine trichodactylid species of two subfamilies and three tribes (Table 1). The observations were made under stereoscopic microscope and scanning electron microscope (SEM). The locations and cephalothorax width and length of each specimen are listed in Table 1.

**Table 1:** List of species observed with SEM in the present study according to their catalog number, sample locations and biometric data.

	Species	Catalog number	CW/CL (mm)
Dilocarcininae tribe Dilocarcinini	*Dilocarcinus pagei* (Stimpson, 1981)	DP-4	31.2/24.9
	*Dilocarcinus septemdentatus* (Herbst, 1783)	INPA-800	27.4/22.8
	*Poppiana argentiniana* (Rathbun, 1905)	PASJ-1	22.4/23.1
Dilocarcininae tribe Valdiviini	*Zilchiopsis collastinensis* (Pretzman, 1968)	ZC-22	43.5
	*Sylviocarcinus australis* (Magalhães & Türkay, 1996)	SAB-19	27.8/25.7
	*Sylviocarcinus devillei* (Milne-Edwards, 1853)	INPA-SD-03	40.9/37.2
Trichodactylinae	*Trichodactylus borellianus* (Nobili, 1896)	RTTB-1	10.7
	*Trichodactylus kensleyi* (Rodríguez, 1992)	TKST-1	13.9
	*Trichodactylus fluviatilis* (Latreille, 1828)	INPA-TF-10	23.5/21.0

**Notes.**

CW cephalothorax width CL cephalothorax length

### Preparation of feeding structures for light and scanning microscope observation

The cephalothorax of crabs were opened and stomachs were manually dissected under stereoscopic microscope. Gut content, coarse food remains and debris were washed with water with the aid of a squeeze bottle. To remove muscular remains and finely attached debris, feeding structures were cleaned in 10% KOH during 24 h, washed with distilled water and conserved in alcohol 70%.

**Figure 1:**
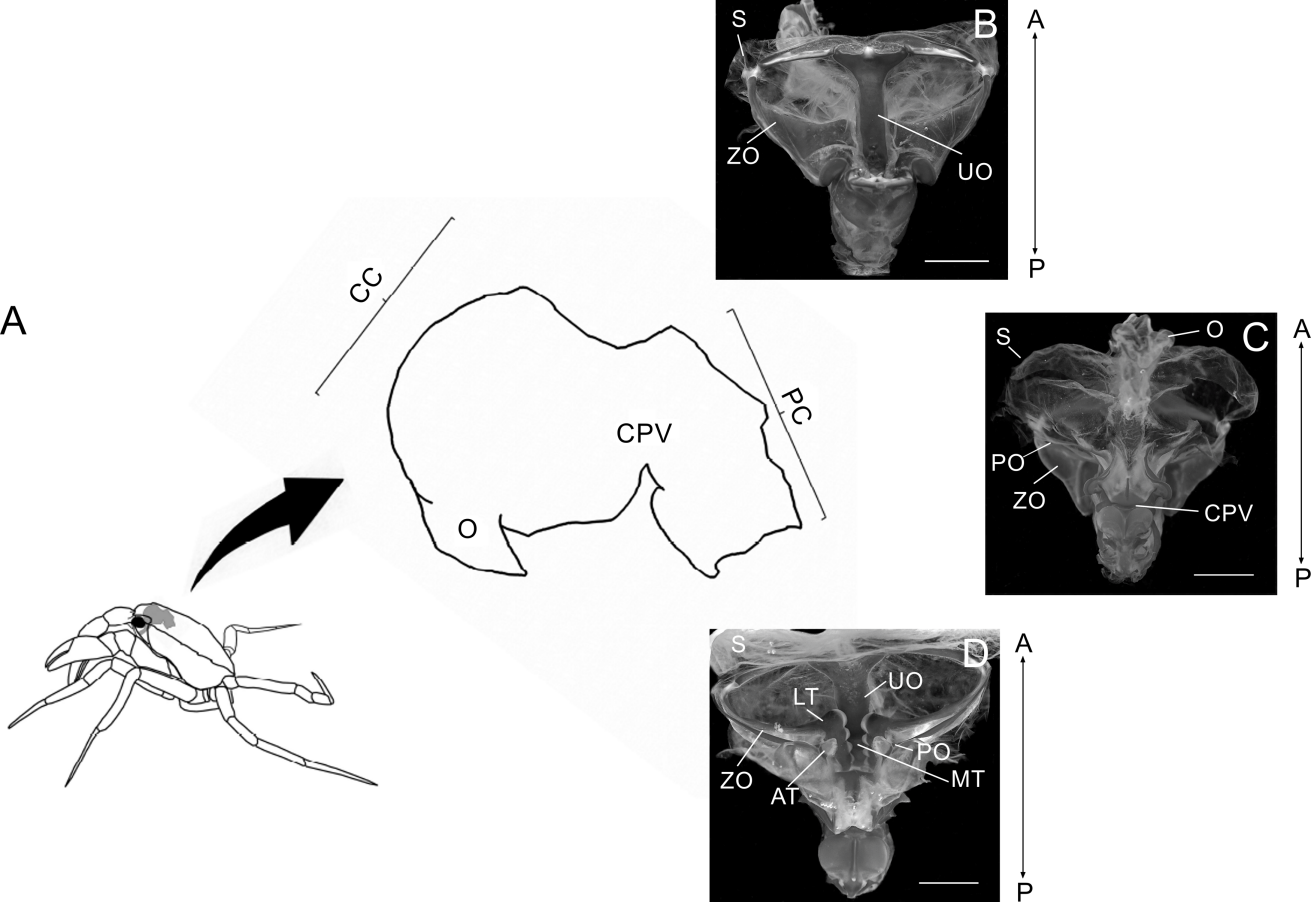
Schematic image showing laterally the stomach in a crab. Schematic image showing laterally the localization of the stomach in a crab, the esophagus (O), cardiac chamber (CC), pyloric chamber (PC) and the cardiopyloric valve (CPV) (A). Light micrograph of the stomach of a *Z. collastinensis* showing the general localization of each studied ossicle (B–C). Dorsal (B) and dorsal (C) views of the stomach showing the sac and ossicles externally. Ventral view of the stomach without sac exposing the ossicles and teeth involved in the food maceration (D). A, Anterior; AT, Accessory teeth; CPV, Cardiopyloric valve; LT, Lateral teeth; MT, Medial tooth; P, Posterior; S, Stomach sac; UO, Urocardiac ossicle; ZO, Zigocardiac ossicles. All scale bars = 5,000 µm.

Each dissected stomach was opened cutting the cardiac sac and exposing the gastric mill. The stomachs used for visualization under light microscope were stained with Alizarin Red 1% after the treatment with KOH 10%. Stomachs were submerged in a recipient with alcohol 70% and photographed with a Canon EOS Rebel T2i camera attached to a Leica S8APO dissecting microscope. Specific images were taken from the ossicles of the cardiac chamber: zigocardiac ossicles, uropyloric ossicle, pectineal ossicle and the cardiopyloric valve. Special emphasis was made on the lateral teeth, medial tooth, accessory teeth and the anterodorsal surface of the cardiopyloric valve (Fig. 1). The terminology used in the foregut descriptions followed those used by Meiss & Norman (1977). Dissected stomachs for analysis with scanning electron microscopy (SEM) were air dried for a minimum of 48 h in a desiccator containing silica gel with an indicator of dampness. Once mounted on a metal stub using double-sided tape and/or silver paint, samples were gold coated for 120 s using Combined Deposition System metal/carbon, SPI Supplies, AX-12157, operated under argon atmosphere (18 mA) for 120 s. Then, observations were made using a JSM-35C scanning electron microscope (JEOL, Shanghai, China), equipped with a system of digital image acquisition SemAfore, at an accelerating voltage of 20 kV.

### Analysis of morphological traits

The morphological traits were encoded into categorical characters as listed in Table 2 according to those observed in the gastric ossicles (Table 3). The generated matrix was analyzed with a hierarchical clustering analysis with unweighted pair-group average using the Euclidean distance in Past 3.16 (Hammer, Harper & Ryan, 2001). The results obtained were compared with the systematic classification of Magalhães & Türkay (1996). The observations from morphological trait were contrasted with the natural diet of the trichodactylid species (*T. borellianus*, *T. kensleyi*, *T. fluviatilis*, *Zilchiopsis oronensis* (Pretzmann, 1978), *D. pagei*) with available information in the literature (Table 4). *Zilchiopsis collastinensis* was included in this case as a representative of the genus *Zilchiopsis*.

**Table 2:** Categorical characters of the morphological traits of ossicles used in the clustering analysis.

Lateral teeth		Medial tooth		Accessory teeth		Cardiopyloric valve	
(1) Surface of the lateral teeth	0-Flattened cusps 1-Incisor cusps 2-Sharp cusps	(3) Shape of medial tooth	0-Posterior process without transverse ridges 1-With less than two transverse ridges 2-With at least three pronounced transverse ridges	(5) Shape of the accessory teeth	0-Vestigial 1-More than 1 softly cuspidate tooth	(7) Ventro-lateral surface	0-W-shaped 1-Smooth
(2) Hollow between ventral and dorsal cusps	0-Absence 1-Presence	(4) Margen ventro-lateral	0-Absence of lateral cusps anterior to the medial tooth 1-Presence of lateral cusps anterior to the medial tooth	(6) Disposal of denticles of the lateral tooth	0-In a row 1-In a semicircle 2-With a single tooth		

**Table 3:** Morphological traits observed in the gastric teeth of Trichodactylidae species. The data about the natural diet of each species refers to the most important trophic resource (animal or vegetal) used by each species.

	Species	Medial tooth	Lateral teeth	Accessory teeth	Cardiopyloric valve	Diet
Dilocarcininae tribe Dilocarcinini	*Dilocarcinus pagei*	Marked transverse ridges (4–5) Lateral spines present	Flattened	Vestigial	W-shaped Pronounced with two ridges	Greater importance of plant items
	*Dilocarcinus septemdentatus*	Marked transverse ridges (3) Lateral spines present	Slightly cuspidate (8)	Vestigial	W-shaped Faint without ridges	–
	*Poppiana argentiniana*	Marked transverse ridges (3) Lateral spines present	Flattened (7)	Vestigial	W-shaped Pronounced without ridges	–
Dilocarcininae tribe Valdiviini	*Zilchiopsis collastinensis*	Faint transverse ridges (3) Lateral spines present	Flattened (7–8)	4 softly cuspidate teeth	–	–
	*Sylviocarcinus australis*	Transverse ridges screw-like (3)	Incisor cusps (7–8)	3 softly cuspidate teeth	W-shaped Pronounced without ridges	–
	*Sylviocarcinus devillei*	Transverse ridges screw-like (4)	Incisor cusps (9–10)	Vestigial	W-shaped Faint without ridges	–
Trichodactylinae	*Trichodactylus borellianus*	Without transverse ridges Cubical and flattened Lateral spines present	Sharp cusps (4–5) Medial hollow	Vestigial 1 softly cuspidate teeth	Smooth	Both animal and vegetable items
	*Trichodactylus kensleyi*	Without transverse ridges Cubical and flattened Lateral spines present	Sharp cusps (4–6) Medial hollow	Vestigial 1 softly cuspidate teeth	Smooth	Both animal and vegetable items
	*Trichodactylus fluviatilis*	Without transverse ridges Cubical and flattened Lateral spines absent	Sharp cusps (4–6) Medial hollow	Vestigial 2-3 softly cuspidate teeth	Smooth	Both animal and vegetable items

**Table 4:** List and frequencies of food items register in stomach content of trichodactylid crabs based on previous studies (* low frequency; ** medium frequency; *** high frequency).

Species/ Food item		Dilocarcininae						Trichodactylinae		
	Dilocarcinini				Valdiviini					
	*D. pagei* (1, 2)	*D. septemdentatus*	*P. argentiniana*	*Z. oronensis* (3)	*Z. collastinensis*	*S. australis*	*S. devillei*	*T. borellianus* (4, 5)	*T. kensleyi* (6)	*T. fluviatilis* (7, 8, 9)
VegR	***			***				***	***	***
Alg	**			*				*	**	***
Fungi	*			–				*	*	–
MicrO	**			*				*	*	–
MicrC	*			–				**	*	–
Olig	*			–				***	***	–
ChrL	*			*				***	**	–
InstL	*			**				***	*	**
Mol	*			–				*	–	*
Anura	–			–				–	–	**

**Notes.**

VegR vegetal remains Alg filamentous and unicellular algae MicrO microorganism (amoebas, ciliates, tardigrades) MicroC microcrustaceans (copepods, cladocerans and rotifers) Olig Oligochaetes ChrL Chironomidae larvae InstL insect larvae Mol mollusks Anura Anura-Hylidae

(1) Williner & Collins (2002); (2) Torres, Giri & Williner (2012); (3) V Williner (2010, unpublished data); (4) Williner & Collins (2013); (5) Carvalho (2014); (6) Williner, Carvalho & Collins (2014); (7) Segadilha & Silva-Soares (2015); (8) Costa et al. (2016); (9) Nogueira-Costa et al. (2016). The gray shaded area indicates the species with unknown feeding habit.

## Results

### General considerations of the gastric mill of Trichodactylidae

The foregut of the studied trichodactylid crabs was equivalent with the general morphology of Brachyura. The gastric mill was a chewing apparatus formed by ossicles with chitin-covered teeth and other ossicle with supporting functions. These chitin-covered teeth protruded from the unpaired urocardiac ossicle and from the paired zigocardiac and pectineal ossicles. These structures, plus the cardiopyloric valve composed the chewing apparatus (Fig. 1). Despite the common features in the morphology of the gastric ossicles of the studied species, the morphology of the gastric teeth was different in each species.

### Similarities and differences of the gastric mill of Trichodactylidae

#### Lateral teeth

**Figure 2:**
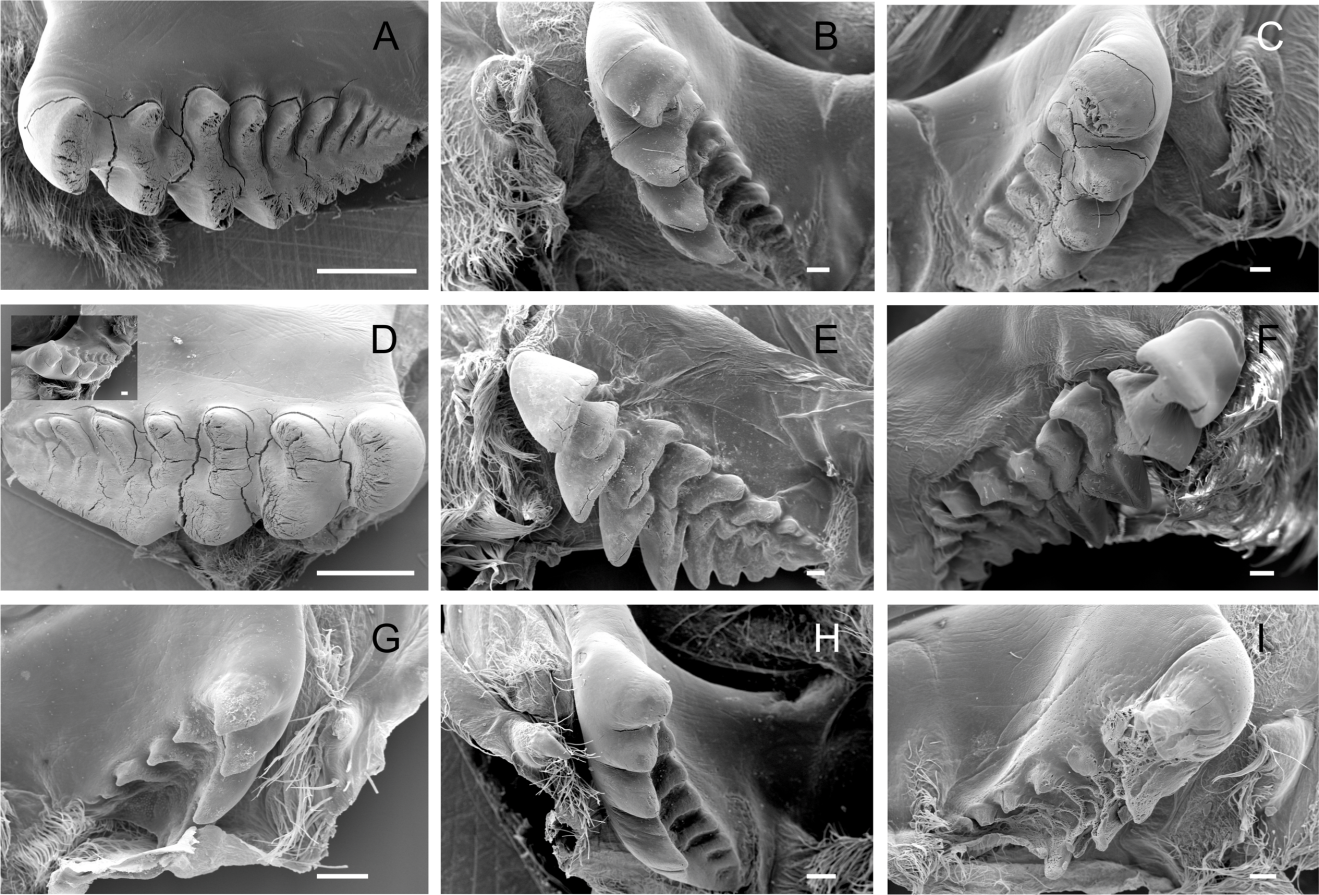
Scanning electron micrographs of the lateral teeth of Dilocarcininae (A–F) and Trichodactylinae (G–I) species. Ventral view of the lateral teeth showing blunt-like cusps of *D. pagei* (A), *D. septemdentatus* (B), *P. argentiniana* (C) and *Z. collastinensis* (D and insert) and incisor-like cusps of *S. australis* (E), *S. devillei* (F). In Trichodactylinae, lateral teeth (ventral view) exhibited ventral and dorsal cusps with sharp-edges separated by a pronounced hollow in *T. borellianus* (G), *T. kensleyi* (H) and *T. fluviatilis* (I). All scale bars = 100 µm.

The lateral tooth rises from the zigocardiac ossicle, a paired structure that extends ventrally and medially into the cardiac stomach. It is the major masticatory structure of the gastric mill. The lateral teeth of the studied trichodactylid species were strongly chitinized and calcified with a variable shape and number of cusps (Figs. 2A–2I) (Table 3). In Dilocarcininae, cusps were blunt or incisor-like (Figs. 2A–2F). *D. pagei*, *D. septemdentatus, Poppiana argentiniana* and *Z. collastinensis* were morphologically similar with blunt-like cusps that decreased in width posteriorly (Figs. 2A–2D). Juvenile or newly molted specimens exhibited more prominent and less worn cusp, such as a *Z. collastinensis* juvenile (Fig. 2D insert) and *P. argentiniana* (Fig. 2B). In *Sylviocarcinus autralis* and *S. devillei*, cusps had edges that were more incisive with intercalated dorsal and ventral cusps (Figs. 2E and 2F). In Trichodactylinae species, lateral teeth were quite different from Dilocarcininae because both ventral and dorsal cusps had sharp edges with a pronounced hollow between them (Figs. 2G–2I). Cusps also reduced in width posteriorly.

#### Medial tooth

**Figure 3:**
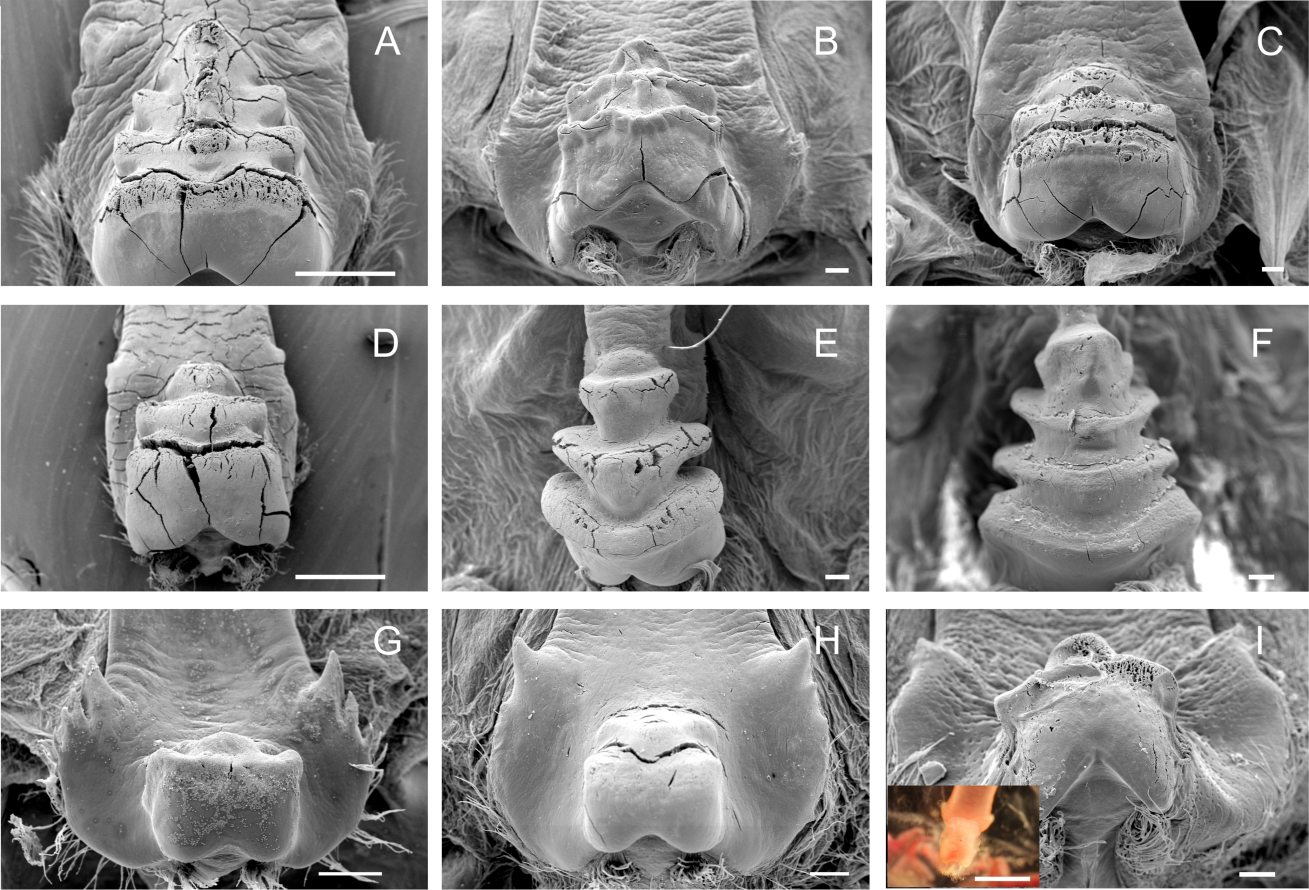
Scanning electron micrographs and light micrograph of the medial tooth of Dilocarcininae (A–F) and Trichodactylinae (G–I) species. Ventral view of the medial tooth showing the transverse ridges of *Dilocarcinus pagei* (A), *D. septemdentatus* (B), *Poppiana argentiniana* (C) and *Zilchiopsis collastinensis* (D). *Silviocarcinus australis* (E) and *S. devillei* (F) exhibited a screw-like tooth. In Trichodactylinae, the medial tooth was characterized by a cubical and smooth process (ventral view) in *T. borellianus* (G), *T. kensleyi* (H) and *T. fluviatilis* (I). All scale bars = 100 µm.

The medial tooth rises at the posterior end of the urocardiac ossicle, an unpaired structure located in the dorsal midline of the cardiac stomach. The medial tooth of trichodactylid species was strongly chitinized and calcified with variable morphology (Figs. 3A–3I) (Table 3). In Dilocarcininae, *D. pagei*, *D. septemdentaus*, *P. argentiniana* and *Z. collastinensis* presented the medial tooth with transverse ridges that increased in size posteriorly with variable number of ridges according to the species (Figs. 3A–3D) (Table 3). In *D. pagei* and *D. septemdentatus*, these transverse ridges were more pronounced than in *P. argentiniana* and *Z. collastinensis* (Figs. 3A–3D). *Sylviocarcinus australis* and *S. devillei* were the most different species within Dilocarcininae, with a screw-like medial tooth with ridges that increased in size posteriorly (Figs. 3E and 3F). In Trichodactylinae, a cubical and blunt/smooth process with slight differences among species (Table 3) characterized the medial tooth. Moreover, laterally to the process, all species exhibited at least a pair of well-defined sub-terminal denticles (Figs. 3G–3I).

#### Accessory teeth

**Figure 4:**
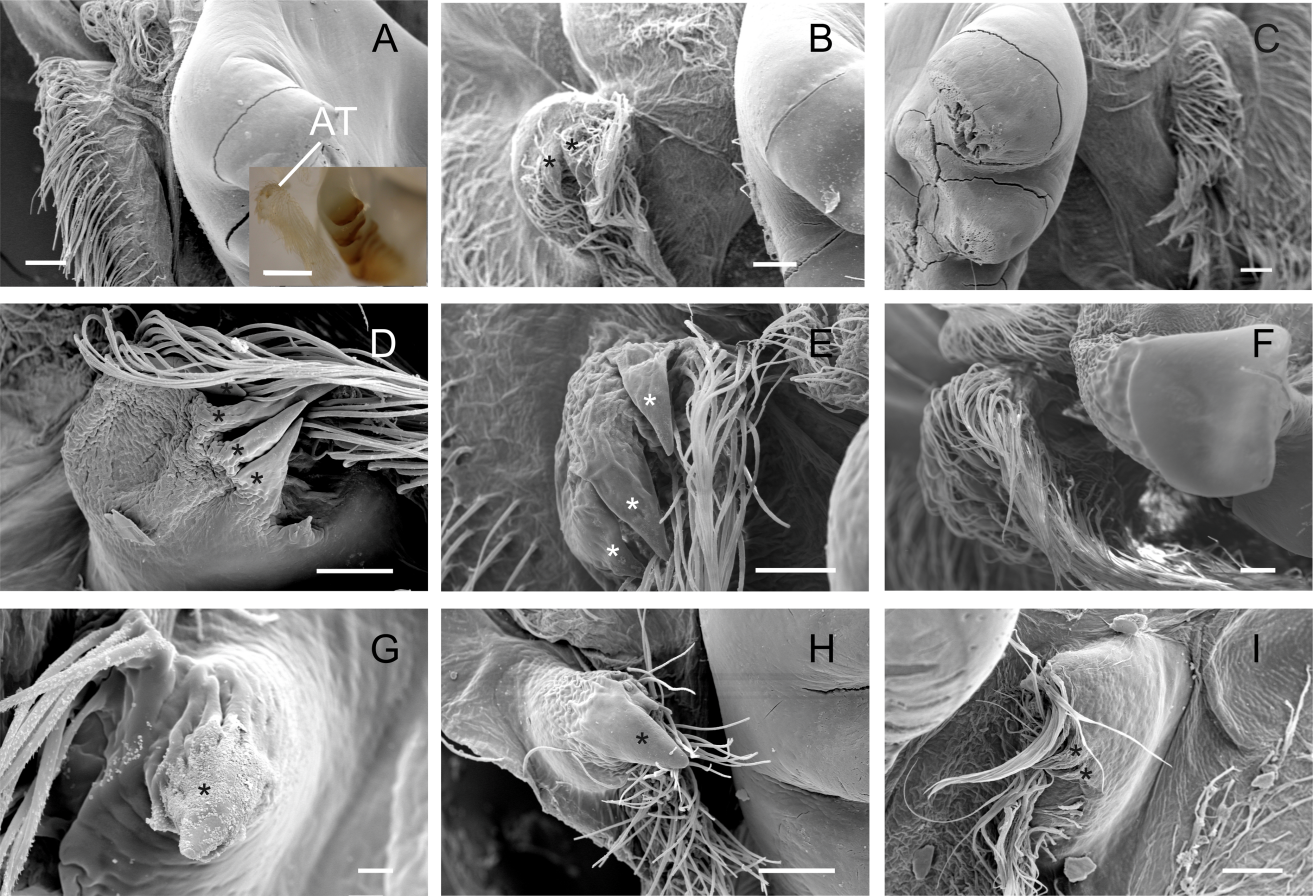
Scanning electron micrographs and light micrograph of the accessory teeth of Dilocarcininae (A–F) and Trichodactylinae (G–I) species. Ventral view of the accessory teeth showing the little chitinization of this structure surrounded by large setae in *D. pagei* (A), *D. septemdentatus* (B), *P. argentiniana* (C), *Z. collastinensis* (D), *Silviocarcinus australis* (E) and *S. devillei* (F). In *T. borellianus* (G) and *T. kensleyi* (H) the accessory tooth (ventral view) has one denticle while in *T. fluviatilis* (I) at least three denticles were observed. Scale bars: A–F = 100 µm (insert A: 1,000 µm). *G* = 10 µm. H–I = 100 µm.

The accessory tooth rises from the pectineal ossicle, a paired structure located laterally in the cardiac stomach. In general, this was a tiny-sized structure moderately to slightly calcified in the studied Trichodactylidae specie. These features could affect the proper viewing because the accessory tooth might not conserve its original form after the preparation stages for SEM. The number of denticles varied according to the species (Table 3) although, in general, large groups of setae surrounded the accessory teeth also hindering the visualization. In Dilocarcininae, this structure seemed to be a bit more robust compared to Trichodatylinae (Figs. 3A–3F), with at least three denticles. Within this subfamily, the accessory tooth was very small in *T. borellianus* and *T. kensleyi* with only one prominent denticle (Figs. 4G and 4H). In *T. fluviatilis*, this structure exhibited at least three visible denticles (Fig. 4I).

#### Cardiopyloric valve

**Figure 5:**
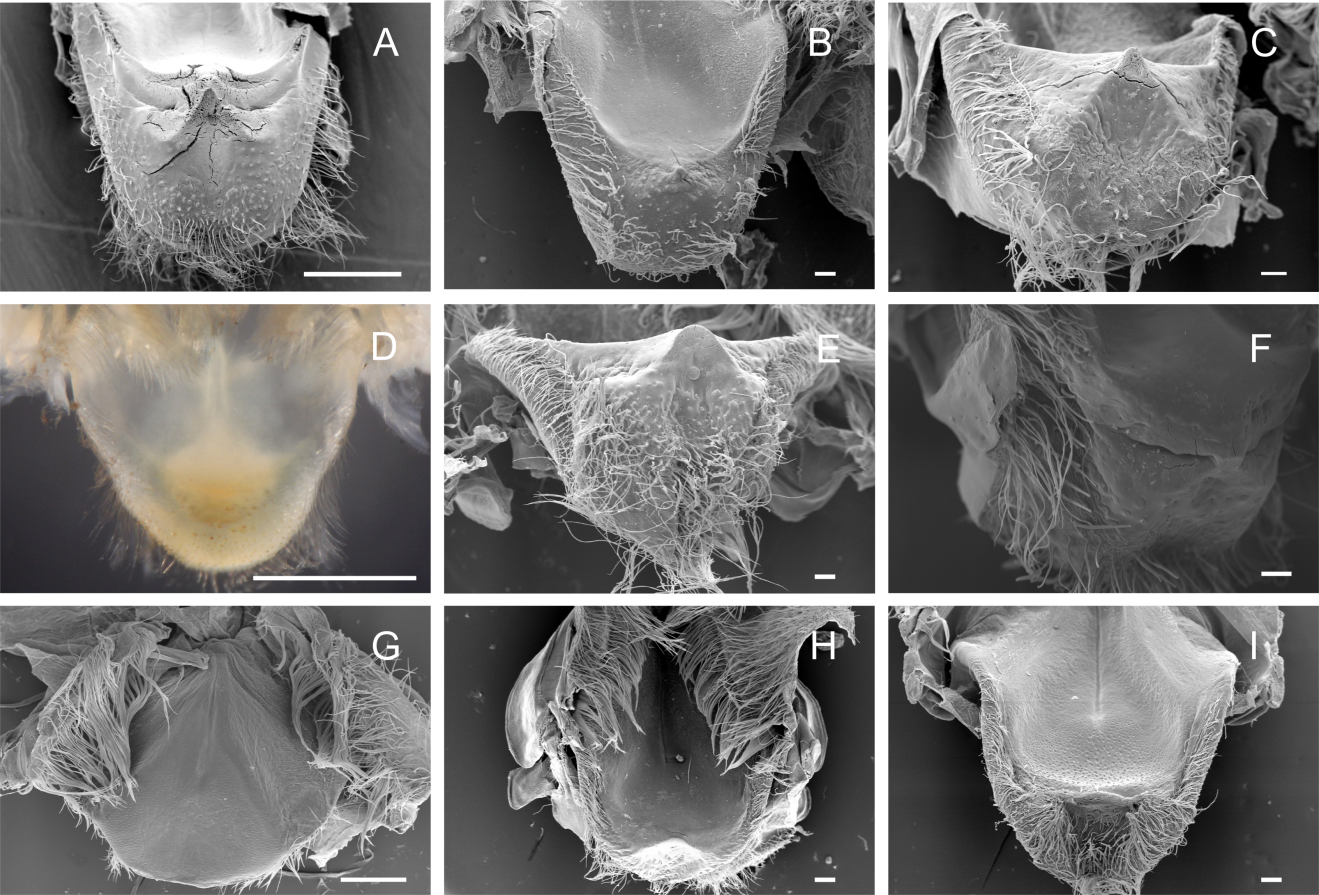
Scanning electron micrographs and light micrograph of the cardiopyloric valve of Dilocarcininae (A–F) and Trichodactylinae (G–I) species. Anterodorsal surface of the cardiopyloric valve showing large setae and a W-design in *D. pagei* (A), *D. septemdentatus* (B), *P. argentiniana* (C), *S. australis* (E) and *S. devillei* (F). In *Zilchiopsis collastinensis* (D) it was only possible to observe the large setae. The cardiopyloric valve of *Trichodactylus borellianus* (G) exhibited a smoother surface while in *T. kensleyi* (H) and *T. fluviatilis* (I) the anterodorsal edge had a concave region. Scale bars: A and D = 1,000 µm. B–C and E–I = 100 µm.

The cardiopyloric valve was located in the ventral midline of the stomach, in the transition of the cardiac and pyloric chambers. The anterodorsal edge of this structure comprised the encounter of the gastric mill and the entrance of the pyloric chamber with the valve. This region had a semicircle shape with numerous setae surrounding the anterodorsal and lateral borders and without tooth-like structures in all species (Figs. 5A–5I) (Table 3). However, valves differed in shape and relief among species. In Dilocarcininae, the anterodorsal surface of the valve of five studied species exhibited a W-design (Figs. 5A–5F) (Table 3). This notwithstanding, in *Z. collastinensis* it was not possible to confirm this trait in stereoscopic microscope due to the level of resolution (Fig. 5D). In *D. pagei*, this morphology was more pronounced, with the presence of two ridges of similar shape (Fig. 5A). In Trichodactylinae, there was no defined W-design (Figs. 5G–5I) but a concave region with less setae in *T. kensleyi* and *T. fluviatilis* (Figs. 5H–5I). In *T. borellianus*, the anterodorsal edge of the valve had less setae and smoother surface compared to Dilocarcininae (Fig. 5G).

#### Morphological traits

**Figure 6:**
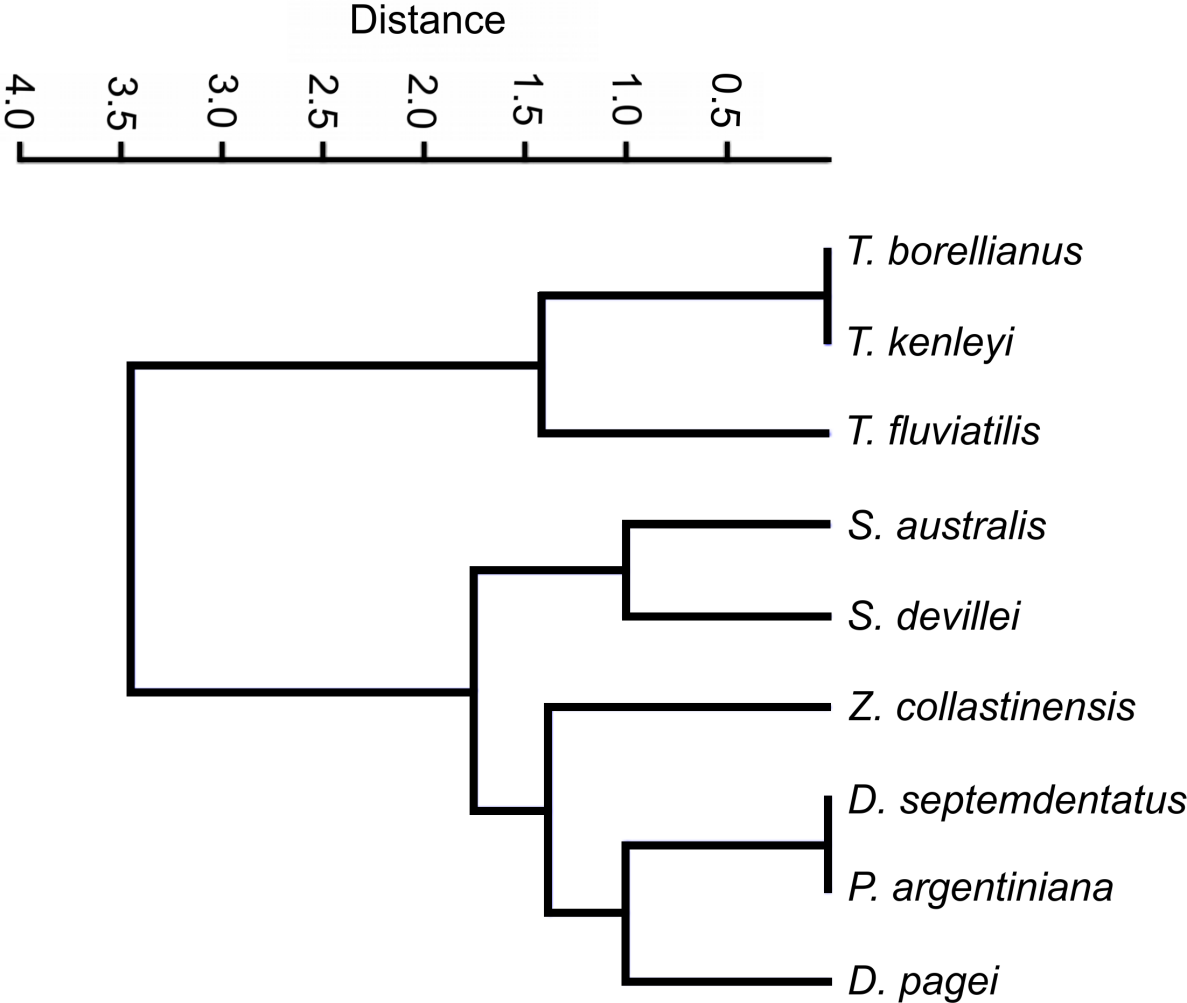
Dendrogram of the hierarchical clustering analysis using categorical characters of the analyzed gastric ossicles of Trichodactylidae species.

The clustering analysis calculated with categorical characters of the observed ossicles revealed that, in general, the species were grouped according the systematic classification of Magalhães & Türkay (1996) with a coefficient of correlation of 0.9775. Only *Z. collastinensis* was clustered more closely to the species of Dilocarcinini tribe (Fig. 6) in contrast the observations of the mentioned authors.

## Discussion

Overall, the analyzed gastric ossicles of trichodactylids were consistent among closely related species. These similarities were more evident among species of the same genus as *Dilocarcinus*, *Sylviocarcinus* and *Trichodactylus*, suggesting that the traits observed in these ossicles had a clear phylogenetic component. These results provide partial evidence to studies that argue that the gastric ossicles have a stronger phylogenetic component than the trophic aspects (Caine, 1975; Felgenhauer & Abele, 1983; Felgenhauer & Abele, 1985; Felgenhauer & Abele, 1989; Brösing & Türkay, 2011). Despite the importance of the phylogenetic component, the relation between feeding habits and morphological traits of the analyzed ossicles matched well in trichodactylid species (*D. pagei*, *T. borellianus*, *T. kensleyi* and *T. fluviatilis*) with known trophic spectra (Williner & Collins, 2002; Williner & Collins, 2013; Williner, Carvalho & Collins, 2014; Carvalho, 2014; Segadilha & Silva-Soares, 2015; Costa et al., 2016; Nogueira-Costa et al., 2016). This result supports our prediction and leads us to speculate that the gastric ossicles involved in the food maceration could be a useful predictor of the feeding habits in those species with unknown natural diet.

### The gastric ossicles of Dilocarcininae: morphological considerations

The gastric ossicles of *Dilocarcinus* spp., *P. argentiniana* and *Z. collastinensis* coincided in many features such as blunt-like cusps in the lateral teeth, transverse ridges in the medial tooth and the cardiopyloric valve with a W-shaped surface. Some slight differences among species were observed in the number and sharpness of cusps and transverse ridges. The morphology of the gastric ossicles of these species was different from *Sylviocarcinus* spp., which exhibited sharper cusps in the lateral teeth and a screw-like medial tooth. Alves, Abrunhosa & Lima (2010) also reported a screw-shaped urocardiac ossicle for *S. pictus* but did not present any illustration or image. Despite of specific dissimilarities, we found that these morphological traits resembled, overall, the gastric ossicles of other herbivorous crabs (Warner, 1977; Giddins et al., 1986; Allardyce & Linton, 2010). The trophic spectra found in the stomach content of *D. pagei* and *Z. oronensis* support the relation between morphological traits and a predominantly herbivorous diet.

These two trichodactylid species (*D. pagei* and *Z. oronensis*) incorporate an important percentage of plant material into their diet, besides some animal items and algae (Williner & Collins, 2002; V Williner, 2010, unpublished data) (Table 3). A diet enriched with vegetal remains requires a gastric mill with structures capable to disrupt the cellulose and hemicellulose. The cusps of the lateral teeth and the transverse ridges of the medial tooth of herbivorous crabs were significantly more pronounced than in species with omnivorous or carnivorous feeding habits (Warner, 1977; Giddins et al., 1986; Allardyce & Linton, 2010). The land crabs *Gecarcoidea natalis* (Pocock, 1888) and *Discoplax hirtipes* Lamarck, 1818 and the mangrove crab *Neosarmatium smithi* (Milne-Edwards, 1853) (cf. Giddins et al., 1986) had a predominant herbivorous diet exhibiting hooked spines in the dorsal cusps of the lateral teeth and transverse ridges in the medial tooth. In trichodactylid crabs, the hooked spines were absent and the other features were less pronounced. Despite the fact that decomposed vegetal remains are actually the main ingested food item in both groups (Greenaway & Linton, 1995; Giddins et al., 1986; Greenaway & Raghaven, 1998; Williner & Collins, 2002; V Williner, 2010, unpublished data), comparison should be made with criteria once both Trichodactylidae and Grapsoidea have different food availability and phylogenetic history (Martin, Crandall & Felder, 2009).

The other ossicles that compose the gastric mill, the cardiopyloric valve and the accessory teeth, seem to be simple structures compared to other decapod species (Caine, 1975; Kunze & Anderson, 1979; Williner, 2010; Brösing & Türkay, 2011). The cardiopyloric valve of Dilocarcininae exhibited a more or less pronounced W-like surface while the accessory teeth is a structure less developed than in the herbivorous crabs: *G. natalis*, *D. hirtipes*, and *N. smithi*. Both structures were previously described to retain the food in the cardiac chamber (Caine, 1975). However, the cardiopyloric valve may also act as a masticatory structure (Caine, 1975) while the accessory tooth may assist to push material into the gastric mill (Kunze & Anderson, 1979). Functional comparison with other brachyuran crabs requires further information of these structures in species with known natural diet.

### The gastric ossicles of Trichodactylinae: morphological considerations

In Trichodactylinae, the overall morphology of the gastric mill was very similar among the observed species and quite different compared to that of Dilocarcininae. Species of the genus *Trichodactylus* analyzed in the present study exhibited lateral teeth with cuspidate cusps separated by a pronounced hollow and a medial tooth with a cubical and smooth process with well-defined sub-terminal denticles. These traits are quite specific of Trichodactylinae with some coincidences with other omnivorous and carnivorous crabs: *Nectocarcinus tuberculosus* Milne-Edwards, 1860 (cf. Salindeho & Johnston, 2003), *Epixanthus dentatus* (White, 1847) (cf. Cannicci et al., 1998), (Skilleter & Anderson, 1986; Heeren & Mitchell, 1997; Cannicci et al., 1998; Salindeho & Johnston, 2003; Allardyce & Linton, 2010). The observed morphological traits of the three *Trichodactylus* spp. analyzed in this study match well with an omnivorous trophic spectra previously described in the literature: *T. borellianus* (Williner & Collins, 2013; Carvalho, 2014), *T. kensleyi* (see Williner, Carvalho & Collins, 2014) and *T. fluviatilis* (Segadilha & Silva-Soares, 2015; Costa et al., 2016; Nogueira-Costa et al., 2016).

*Trichodactylus* spp. exhibit a natural diet with great importance of both vegetal and animal components (Williner & Collins, 2013; Carvalho, 2014; Williner, Carvalho & Collins, 2014; Segadilha & Silva-Soares, 2015; Costa et al., 2016; Nogueira-Costa et al., 2016). An efficient exploitation of these trophic resources requires a gastric mill capable of grinding soft material and also disrupting fibrous structures. The blunt medial tooth observed in *Trichodactylus* spp. revealed great similarity with the omnivorous rock crab *N. tuberculosus* (Salindeho & Johnston, 2003) and the carnivorous xanthoid crab *E. dentatus* (Cannicci et al., 1998). This notwithstanding, the lateral teeth of *Trichodactylus* spp. exhibited ventral and dorsal cuspidate cusps of similar morphology, whereas in others carnivorous and omnivorous crabs (*E. dentatus*, *Geograpsus grayi* Milne-Edwards, 1853, *G. crinipes*, *Ozius truncatus* Milne Edwards, 1834, *Leptograpsus variegatus* (Fabricius, 1793), *N. tuberculosus, Pseudocarcinus gigas* (Lamarck, 1818)), the dorsal surface is a ridge-like structure (Skilleter & Anderson, 1986; Heeren & Mitchell, 1997; Cannicci et al., 1998; Salindeho & Johnston, 2003; Allardyce & Linton, 2010).

In coincidence with what was observed in Dilocarcininae, the cardiopyloric valve and the accessory teeth of Trichodactylinae were simple structures compared to anomurans (*Dardanus setifer* (Milne-Edwards) (Kunze & Anderson, 1979); *Clibanarius vittatus* (Bosc), *Petrochirus diogenes* (L.) (Caine, 1975); *Aegla uruguayana* (Williner, 2010)), hermits crabs (*Clibanarius taeniatus* (Milne Edwards) (Kunze & Anderson, 1979)), and brachyuran (*Discoplax gracilipes* Ng & Guinot, 2001, *Cardisoma armatum* Herklots, 1851, *Epigrapsus notatus* (Heller, 1865) (Lima, 2010)). Comparing both subfamilies, the cardiopyloric valve of Trichodactylinae had a smoother anterodorsal surface. If this structure is involved in the food maceration, the absence of a W-shaped surface coincides with a diet with less importance of vegetal items than Dilocarcininae. The accessory teeth were a tiny-size structure in both subfamilies and seem to be less developed in Trichodactylinae. However, this structure was different from other crabs with varied trophic habit, possibly due to this morphological trait being a trichodactylid-owned feature, as per our findings and the mentioned literature.

### Comments on phylogenetic and functional aspects of gastric ossicles

Our results, together with the information available in the literature about the morphology of the gastric armature, strongly support the hypothesis that this structure conserves much of the phylogenetic history of a taxon (Felgenhauer & Abele, 1989; Brösing, Richter & Scholtz, 2006; Brösing, 2010; Brösing & Türkay, 2011). However, and as mentioned by Brösing & Türkay (2011): “The morphology of the foregut ossicles and the attached gastric teeth have to be evaluated separately”. Despite being evident that the gastric ossicles have a conservative pattern (e.g., number of ossicles) even being used in the construction of phylogenies (Felgenhauer & Abele, 1989; Brösing, 2010), it is striking that species phylogenetically distant and with similar trophic habit exhibit similar morphological traits of the gastric teeth (Schaefer, 1970; Caine, 1975; Dall & Moriarty, 1983; Felgenhauer & Abele, 1985; Felgenhauer & Abele, 1989; Allardyce & Linton, 2010; Brösing & Türkay, 2011).

In the Trichodactylidae species, we were able to identify morphological traits of the gastric teeth that were, overall, similar to that of other phylogenetically distant crabs of similar trophic habit. The transverse ridges, typical of herbivorous crabs such as *G. natalis*, *D. hirtipes* (cf. Allardyce & Linton, 2010) and *N. smithi* (cf. Giddins et al., 1986), were present in all analyzed Dilocarcininae species with variable number and ridges in the medial tooth and cardiopyloric valve. The stomach content analysis of *D. pagei* and *Z. oronensis* revealed that these species ingested predominantly vegetal material (Williner & Collins, 2002; V Williner, 2010, unpublished data), as could be expected based on the gastric mill. In Trichodactylinae, the species exhibited blunt and smooth structures (the medial tooth and the cardiopyloric valve) such as those found in *N. tuberculosus* (cf. Salindeho & Johnston, 2003) and *E. dentatus* (cf. Cannicci et al., 1998). These traits are observed in species with less fibrous contents in their diet (Salindeho & Johnston, 2003; Allardyce & Linton, 2010). The three *Trichodactylus* spp. examined in this study presented an omnivorous feeding habit with high frequency of animal items (Williner & Collins, 2013; Williner, Carvalho & Collins, 2014; Carvalho, 2014; Segadilha & Silva-Soares, 2015; Costa et al., 2016; Nogueira-Costa et al., 2016). However, the vegetal remains were also an important trophic resource for *Trichodactylus* spp. Functionally, the cuspidate lateral teeth could supplement the absence of cuspidate structures in the medial tooth and the cardiopyloric valve, aiding in the disruption of fibrous content. These observations provide more examples of crabs that exhibited similar trophic habits and morphological features despite not being closely related species.

Considering the consistent relationship found between the gastric teeth traits of trichodactylid species with known feeding habits, it is plausible to predict that the analyzed Dilocarcininae species with unknown trophic spectra exhibit a predominantly herbivorous diet. Despite the tendency towards more herbivore or carnivorous trophic habits, trichodactylids are generally omnivores (Collins, Williner & Giri, 2007), which suggests that they are capable of finding, capturing, ingesting and digesting prey of different trophic levels and behaviors, morphologies and chemical compositions (Diehl, 2003). This implies that they must have behavioral, sensory, morphological and physiological adaptations that allow them to identify and incorporate into the diet a varied range of foods of both animal and vegetable origins (Eubanks, Styrsky & Denno, 2003). From a morphological perspective, we observed that the stomach of the studied crabs, while showing features more adjusted to an herbivorous or carnivorous diet, presented a morphology that allows the mechanical digestion of a variety of food of animal and vegetable origin. Indeed, the design that we appreciate today must be a trade-off between the ability to gather food items available in the environment and the ability to digest, in order to favor the continuity of that design (Turner, 2007). As mentioned by Stevens & Hume (1995): “In any one taxon, each of these digestive organs will have morphological characteristics that functionally reflect the evolutionary history and contemporary ecology of the taxon”.

Since the continental invasion of trichodactylid ancestors in the post-Gondwanan period (Yeo et al., 2008; Cumberlidge & Ng, 2009), the ability to conquer new freshwater environments was shaped by habitat heterogeneity and complicated topography and hydrology (Yeo et al., 2008). It follows, in this context, that those morphological traits that facilitated an opportunistic diet may be favored (Waloszek et al., 2007). Nowadays, the trophic spectra of trichodactylid crabs exhibit omnivorous and opportunistic trophic habit with variable importance of vegetal and animal items according to the species (Collins, Williner & Giri, 2007; Williner & Collins, 2013; Williner, Carvalho & Collins, 2014; Carvalho, 2014; Segadilha & Silva-Soares, 2015; Carvalho et al., 2016; Costa et al., 2016; Nogueira-Costa et al., 2016). This trophic habitat is in concordance with the availability of trophic resources (Carvalho et al., 2016) typical of large rivers of South America subjected to constant variation due to the high renewal of water of these pulsatile systems (Junk, Bayley & Sparks, 1989; Neiff, 1996).

The digestive capacity of trichodactylid crabs or any other taxon must be counteracted by factors other than morphology to compensate differences in the quality of food. Similarity in diet but pronounced differences in gastric mill morphologies could indicate a partial analysis of what the completely digestive process would be, or indicate different approaches to process the same food. The adjustments to digest a determined food will be constrained by the phenotypic plasticity of a taxon, which in turn depends on the evolutionary time scales changes in the genotype (Linton & Greenaway, 2007; Piersma & Van Gils, 2011). The enzymatic response to specific nutrients of each species (Fernández-Gimenez, 2013), the ingestion of bacteria with the food (Harris, 1993), the feeding preferences (Constantini & Rossi, 2001), are a few examples of complementary factors that must be studied before any solid conclusions can be made about the role of dietary and historical components in shaping morphological traits. Future studies should concentrate on the efforts in quantify the role of phylogeny and function in shaping morphological traits of the gastric ossicle through analysis of phylogenetic signal, defined as the “tendency for related species to resemble each other more than they resemble species drawn at random from the tree” (Blomberg & Garland, 2002).

## Conclusions

Our results revealed that species with similar trophic habits but not closely related (at the family level and even at the infraorder level) exhibited similar morphologies of the gastric teeth. At the family level, this study provides evidence that the morphological traits of the gastric teeth of trichodactylid crabs seem to be well adjusted to the feeding habit of these omnivorous crabs, according to a more vegetal or animal-based diet. This notwithstanding, the relationship between morphological traits and trophic habit is a partial observation that must be analyzed with caution. In this sense, we strongly suggest, whenever possible, the use of phylogenetic signal analysis as a tool for quantifying the role of phylogeny and function in the design of any morphological trait.

## Additional Information and Declarations

### Data Availability

The following information was supplied regarding data availability: The specimens described in the manuscript were stored at: (1) Instituto Nacional de Limnología with the following ID numbers: *D. pagei* DP-4 *P. argentiniana* PASJ-1 *Z. collastinensis* ZC-22 *T. borellianus* RTTB-1 *T. kensleyi* TKST-1. (2) Instituto Nacional de Pesquisas da Amazônia with the following ID numbers: *D. septemdentatus* INPA-800 *S. australis* SAB-19 *S. devillei* INPA-SD-03 *T. fluviatilis* INPA-TF-10.
